# Transfer Irreversibilities in the Lenoir Cycle: FTT Design Criteria with ε−NTU

**DOI:** 10.3390/e27121262

**Published:** 2025-12-18

**Authors:** Ricardo T. Páez-Hernández, Juan Carlos Pacheco-Paez, Juan Carlos Chimal-Eguía, Delfino Ladino-Luna, Javier Contreras-Sánchez

**Affiliations:** 1 Area de Física de Procesos Irreversibles, Departamento de Ciencias Básicas, Universidad Autónoma Metropolitana, U-Azcapotzalco, Av. San Pablo No. 420, Col. Nueva el Rosario, Alcaldía Azcapotzalco, Ciudad de México C.P. 02128, Mexico; dll@azc.uam.mx; 2 Departamento de Ciencias Básicas, Universidad Autónoma Metropolitana, U-Azcapotzalco, Av. San Pablo No. 420, Col. Nueva el Rosario, Alcaldía Azcapotzalco, Ciudad de México C.P. 02128, Mexico; 3 Departamento de Biofísica, Escuela Nacional de Ciencias Biológicas, Instituto Politécnico Nacional, D.R. Instituto Politécnico Nacional (IPN). Av. Luis Enrique Erro S/N, Unidad Profesional Adolfo López Mateos, Zacatenco, Alcaldía Gustavo A. Madero, Ciudad de México C.P. 07738, Mexico; javcontreras@ipn.mx; 4 Laboratorio de Ciencias Matemáticas y Computacionales, Centro de Investigación en Computación, Instituto Politécnico Nacional, Ciudad de México C.P. 07738, Mexico; jchimale@ipn.mx

**Keywords:** finite-time thermodynamics, steady flow Lenoir cycle, heat conductance, efficient power, bounded ecological function, second law efficiency

## Abstract

This work extends the steady flow Lenoir cycle within finite-time thermodynamics (FTT) by incorporating heat transfer irreversibilities through the ε−NTU formalism and a non-isentropic expansion modeled via the expander isentropic efficiency $\eta_{E}$. The total conductance $U_{T}$ (sum for the two heat exchangers) is partitioned between hot and cold units using $u_{L}=U_{L}/U_{T}$, with $U_{T}=U_{H}+U_{L}$. For each triplet ($\tau =T_{H}/T_{L}$, $U_{L}$, $U_{T}$), we closed the cycle by determining $T_{1}$, the working fluid temperature at the cooler outlet and heater inlet, $T_{2}$, the heater outlet and expander inlet, and $T_{3}$, the expander outlet and cooler inlet. Using these states, we compute the heat rates ${\dot{Q}}_{12}$, ${\dot{Q}}_{31}$ and the net power P. In addition to the thermal efficiency η, the following extended objective functions are evaluated: the efficient power $E_{F}$, the ecological efficiency ϕ, and the second law efficiency $\eta_{II}$. Parametric sweeps on $u_{L}$ for $\tau \;\epsilon \;\left\{3.25,3.75\right\}$ and $U_{T\;}\epsilon \;\left\{2.5,5.0,7.5,10\right\}\;kW$ show unimodal curves for P($u_{L}$) and maxima. A robust result places the optima of P, η, $E_{F}$, ϕ, and $\eta_{II}$ in a distribution band at $u_{L}\sim 0.6$. This guideline offers clear design guidance for allocating exchange area in heat recovery and microgeneration, maximizing power, high η, and exergetic utilization with contained entropic penalty.

## 1. Introduction

Finite-Time Thermodynamics (FTT) [1,2,3,4,5] is a branch of thermodynamics developed to analyze irreversible processes in real systems, particularly in power plants and heat engines, where thermodynamic equilibrium is not reached instantaneously. FTT seeks theoretical models that describe the operating limits of these systems under realistic constraints of dissipation, irreversibility, efficiency, and power.

One of the key motivations of FTT is to improve the design and performance of thermal devices by considering not only the ideal cycle efficiency but also other relevant variables such as entropy production, power output, or even functions that balance multiple performance criteria [6,7,8,9,10]. A historical milestone was the Curzon–Ahlborn heat engine [1], which proposed a Carnot engine model irreversibly coupled to its heat reservoirs by finite thermal resistances. The model predicted an efficiency at maximum power that surprisingly coincides with efficiencies observed in real power plants.

Velasco et al. [11] introduced the so-called saving functions, dimensionless compromise criteria that compare a given operating point with a reference one and quantify the saving in fuel consumption or in entropy generation. By simultaneously weighting power output and dissipation, these functions provide a unified way to assess operating regimes beyond the traditional focus on either maximum efficiency or maximum power. Building on this formalism, Barranco-Jiménez et al. [12] applied the saving functions to the Novikov engine [13,14] and analyzed different levels of participation of irreversible processes and considered two alternative heat transfer laws, which made it possible to evaluate the sensitivity of power and efficiency to variations in the model parameters.

The advancement of the FTT has also incorporated new multi-objective criteria, such as the ecological function and efficient power, and both approaches aimed at simultaneously minimizing energy loss and environmental degradation [15,16]. These criteria are fundamental for the energy transition towards cleaner and more efficient technologies and have been applied to a wide variety of endoreversible and irreversible cycle models, including modified Brayton cycles and thermoelectric generators [16,17,18,19,20,21,22,23,24,25,26,27,28,29,30,31,32,33].

In the face of increasing global energy demands and environmental constraints, the optimization of thermodynamic cycles has become a cornerstone of modern energy system design. While the Carnot and Otto cycles have traditionally dominated the landscape of thermodynamic analysis, the Lenoir cycle [34], historically associated with early internal combustion engines, is gaining renewed interest due to its relevance in pulsed detonation engines, fast-acting pneumatic systems, and micro-scale energy conversion devices.

In these contexts, the Lenoir cycle often serves as a simplified model of constant-volume combustion followed by expansion, and the conductance–allocation guidelines developed in this work are directly applicable to the design of heat recovery units and micro-generation systems based on Lenoir-type architectures.

Several authors have already analyzed the Lenoir cycle within irreversible and finite-time frameworks. Georgiou investigated the ideal Lenoir cycle with regenerative preheating, clarifying the impact of regeneration on useful work and thermal efficiency [35]. Shen et al. considered an endoreversible Lenoir cycle coupled to constant-temperature reservoirs, identifying the influence of finite heat transfer rates on performance [36]. Ahmadi et al. carried out a thermo-economic, multi-objective optimization of an endoreversible Lenoir engine, exploring trade-offs between power, efficiency, and economic cost [37]. More closely related to the present work, Rubio and Wang [38] studied an irreversible steady flow Lenoir cycle with external irreversibilities modeled by ε−NTU heat exchangers and internal irreversibilities in the expander and optimized power and thermal efficiency under a fixed (symmetric) allocation of the total thermal conductance between the hot and cold exchangers. Building on this framework, the present paper focuses on a steady flow Lenoir cycle with explicit ε−NTU heat exchangers and a finite isentropic efficiency of the expander and extends Rubio and Wang’s model by allowing the total thermal conductance to be distributed through an allocation parameter φ and by comparing several objective functions (power, efficient power, bounded ecological function, and second law efficiency) within a unified design framework.

**Figure 1:**
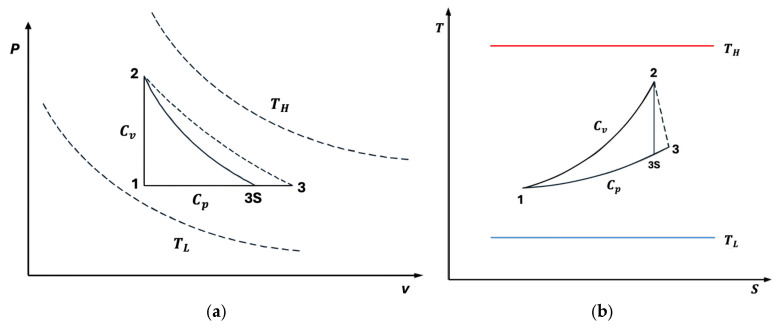
(**a**) p−v  diagram for the irreversible Lenoir cycle. (**b**) T−s diagram for the irreversible Lenoir cycle.

The classical Lenoir cycle comprises three fundamental processes: a constant-volume heat addition $\left(1\to 2\right)$, an adiabatic expansion $\left(2\to 3\right)$, and a constant-pressure heat rejection $\left(3\to 1\right)$. In Figure 1a,b, we also present T-s and P-V diagrams of the cycle for better thermodynamic insight. However, real systems inherently deviate from these idealizations due to irreversibilities, such as internal dissipation and finite-rate heat transfer. To better reflect realistic energy conversion performance, irreversible cycle models have been proposed, introducing internal irreversibility parameters and nonlinear heat transfer laws.

As we have mentioned, this work focuses on the optimization of an irreversible steady flow Lenoir cycle following the approach of [34,35,36,37,38] and incorporating the following:

1. Internal irreversibilities represented by an effective isentropic efficiency of the expander.

2. Newtonian heat transfer laws to account for finite thermal gradients.

3. Ecological function ($E_{F}$), which balances power output and entropy generation to address environmental considerations.

4. A second law efficiency $\left(\eta_{II}\right)$, defined as the ratio between the net power output and the thermal exergy rate supplied by the hot source, which quantifies how effectively the available exergy is converted into useful work.

5. Efficient power ($E_{P}$), which optimizes the product of power and thermal efficiency, representing a compromise between quantity and quality of energy conversion.

6. An extended analytical framework for these optimization regimes, together with a detailed numerical example using realistic parameter values.

## 2. Thermodynamic Description of the Irreversible Lenoir Cycle

In this section, we construct the irreversible model of the Lenoir cycle under steady flow conditions. Our goal is to consistently calculate the net power output of the cycle and the turbine efficiency based on design parameters, such as the quality of the heat exchangers and the losses in the expansion stage. To arrive at these expressions, we need to rigorously describe the internal thermodynamic states of the cycle and the energy and entropy balances in each process.

### 2.1. Working Fluid and Thermodynamic Properties

Since the Lenoir cycle we are analyzing operates with a gas, we adopt the ideal gas model with constant specific heats. This means that the specific enthalpy h and the specific internal energy u depend linearly on the temperature such that $h=C_{p}T$ and $u=C_{v}T$, where $C_{p}$ and $C_{v}$ are the specific heats at constant pressure and volume, respectively, and are taken as constant over the operating range. Furthermore, the relationship between these two heat capacities is given by

(1) $$k\equiv \frac{C_{p}}{C_{v}}.$$

This relationship indicates how much work a gas can do when it expands or contracts; it also allows us to easily convert any ∆T jump into an enthalpy or energy jump and allows us to relate temperature and pressure in isentropic processes.

### 2.2. Physical Structure of the Lenoir Cycle in Steady Flow

The cycle we are considering has three main stages, which correspond directly to the classic stages of the Lenoir cycle but in a steady state and with explicit irreversibilities.

Process 1→2 (constant-volume heat addition) is modeled as nearly isochoric heating in a combustion chamber or plenum upstream of the expander, where the working gas is heated from $T_{1}$ to $T_{2}$ at an essentially fixed volume (or fixed vessel geometry).

Process 2→3 (expansion) is where the fluid then expands in a gas turbine or expansion device, producing shaft work. Ideally, this would be an isentropic expansion, but in practice, losses are present and are captured by an isentropic efficiency $\eta_{E}$.

Process 3→1 (constant pressure heat rejection) is where, finally, the fluid is cooled at approximately constant pressure in a heat recovery or exhaust heat exchanger, returning from state 3 to state 1 and closing the cycle.

According to [38], stages 1→2 and 3→1 are explicitly modeled as processes where heat exchange is limited by actual transfer surfaces and is, therefore, irreversible. To describe this thermal irreversibility in a quantifiable way, the authors use the formulation of ε−NTU [38] heat exchangers. Our work follows the same philosophy.

### 2.3. Heat Exchangers with the ε–NTU Model

Let us assume that the cycle receives and rejects heat through two heat exchangers (HX): one “hot” $\left(U_{H}\right)$, which connects the working fluid to a hot heat source at a fixed temperature $T_{H}$, and one “cold” $(U_{L}$), which connects the working fluid to a cold source at a fixed temperature $T_{L}$.

We know that these heat exchangers are not ideal; that is, their heat transfer capacity is limited by their area, convective coefficients, etc. All this complexity is summarized in an overall conductance U. Assuming that the sum of the available conductance is fixed, we obtain the following:

(2) $$U_{T}=U_{H}+U_{L},$$

where $U_{H}$ is the part allocated to the hot side (heating 1→2) and $U_{L}$ is the part allocated to the cold side (rejection 3→1). To analyze how to distribute this “total exchange capacity” between the heater and the cooler, we introduce $U_{H}=\left(1-u_{L}\right)U_{T}$ and $U_{L}=u_{L}U_{T}$, where $u_{L}$ is a fraction between 0 and 1. In this work, the total thermal conductance $U_{T}$ is defined in the usual way as the ratio between a heat transfer rate and a temperature difference, $U_{T}\equiv \dot{Q}/\Delta T$, so its physical dimension is that of power divided by temperature. Since all heat rates and the net power of the cycle are expressed in kilowatts, we consistently take $U_{T}$, $U_{H}$, and $U_{L}$ in $U_{T}$ in kW/K. This is entirely equivalent to using W/K and simply corresponds to a change in scale ($1\;kW/K=10^{3}\;W/K$), but it is more convenient here because it keeps the numerical values of conductances of the same order of magnitude as the power output. Thus, throughout the paper, the units of the overall thermal conductances are kW/K, in accordance with the definition $U\equiv \dot{Q}/∆T$.

In the case where one of the two streams in the exchanger acts as an almost immutable temperature reservoir (i.e., the hot source is maintained at $T_{H}$ or the cold source at $T_{L}$), the so-called thermal effectiveness E of the exchanger can be written as follows:

(3) $$E=1-\exp\left(-NTU\right),$$

The ratio of heat capacities between the streams is very small, which is precisely the hypothesis used in [38]. Here, “NTU” (Number of Transfer Units) is

(4) $$NTU=\frac{U}{\dot{m}C_{cap}},$$

where $\dot{m}C_{cap}$ is the heat capacity rate (or thermal capacity rate) of the stream whose temperature changes. For the heat exchanger (given in 1→2), the fluid absorbs heat with thermal capacity $\dot{m}C_{v}$; then

(5) $$N_{H}=\frac{U_{H}}{\dot{m}C_{v}}=\frac{{\left(1-u_{L}\right)U}_{T}}{\dot{m}C_{v}},$$

(6) $$E_{H}=1-e^{-N_{H}},$$

where $E_{H}$ measures how closely the internal fluid approaches temperature $T_{H}$. In the cold exchanger (given in 3→1), the fluid releases heat at constant pressure, so its relevant capacity is $\dot{m}C_{p}=\dot{m}{kC}_{v}$. We define the following:

(7) $$N_{L}=\frac{U_{L}}{\dot{m}C_{p}}=\frac{u_{L}U_{T}}{\dot{mk}C_{v}},$$

(8) $$E_{L}=1-e^{-N_{L}}$$

These quantities are fundamental because they allow us to go directly from design parameters of the exchanger (area, coefficients, etc.) to cycle temperatures.

During process 1→2, the cycle gas exchanges heat with the hot reservoir at constant temperature $T_{H}$. Because the process is essentially modeled as an isochoric process for the working fluid, the pressure rises, and the temperature increases from $T_{1}$, which is the working fluid temperature at the cooler outlet and heater inlet, to $T_{2}$, which is the heater outlet and expander inlet. The thermal effectiveness $E_{H}$ of the heat exchanger is then defined as follows:

(9) $$E_{H}=\frac{T_{2}-T_{1}}{T_{H}-T_{1}}.$$

In this equation, when $E_{H}=1$, the gas will exit at exactly the temperature of the hot source ($T_{2}=T_{H}$); if $E_{H}=0$, the gas is not heated ($T_{2}=T_{1}$). For any intermediate value of $E_{H}$, the outlet temperature is a linear interpolation; that is

(10) $$T_{2}=T_{1}+E_{H}\left(T_{H}-T_{1}\right).$$

After heating, the fluid expands to produce work (during process 2→3). Before introducing the irreversibilities of the machine, we first construct the ideal state, state 3s, which would be the final state if the expansion were isentropic. For an ideal gas with constant specific heats, a sentropic expansion obeys the relation $Tp^{\frac{1-k}{k}}=constant$. The Lenoir cycle has a useful geometric feature. Process 1→2 is considered isochoric (constant volume), which implies that the relationship between pressure and temperature satisfies p∝T in this process. This implies

(11) $$\frac{T_{2}}{T_{3s}}={\left(\frac{p_{2}}{p_{3s}}\right)}^{\frac{k-1}{k}}={\left(\frac{T_{2}}{T_{1}}\right)}^{\frac{k-1}{k}},$$

therefore, we can write

(12) $$T_{3s}={T_{1}\left(\frac{T_{2}}{T_{1}}\right)}^{\frac{1}{k}}.$$

This is the lowest temperature the gas could reach after expansion, if the expansion were perfectly reversible (without internal losses). In practice, the expansion is not ideal; there are irreversibilities, and the machine does not extract the maximum possible work, introducing this loss through an isentropic efficiency of the expansion, $\eta_{E}$, which is defined as

(13) $$\eta_{E}=\frac{h_{2}-h_{3}}{h_{2}-h_{3s}}=\frac{T_{2}-T_{3}}{T_{2}-T_{3s}}.$$

This definition is standard for expansions in turbomachines. If $\eta_{E}=1$, the expansion is ideal, and the actual outlet temperature $T_{3}$ coincides with $T_{3s}$. If $\eta_{E}<1$, the expansion is worse, and, therefore, $T_{3}$ will be hotter than $T_{3s}$ (work potential is wasted).

We solve for $T_{3}$ from Equation (13) in a direct algebraic way as follows:

(14) $$T_{3}=\left(1-\eta_{E}\right)T_{2}+\eta_{E}T_{3s}.$$

This expression tells us how the mechanical/thermofluid dynamic inefficiency in the expansion stage “raises” the outlet temperature $T_{3}$ with respect to the ideal temperature $T_{3s}$, penalizing the work. After the cycle expansion, the still-hot fluid (state 3 at temperature $T_{3}$) must then be cooled back to the initial state 1 at temperature $T_{1}$. Using the ε−NTU formulation, the cooling heat exchanger’s effectiveness $E_{L}$ is defined as

(15) $$E_{L}=\frac{T_{3}-T_{1}}{T_{3}-T_{L}}.$$

$E_{L}$ is physically entirely parallel to the hot case. If $E_{L}=1$, the fluid would exit at exactly $T_{L}$; if $E_{L}=0$, it would not cool at all. Solving this relationship for $T_{3}$ in terms of $T_{1}$ and $T_{L}$ is performed as follows:

(16) $$T_{3}=\frac{T_{1}-E_{L}T_{L}}{1-E_{L}}.$$

This equation provides information that, given the effectiveness of the cold exchanger, the actual outlet temperature of the expansion $T_{3}$ cannot be arbitrary; it is strongly controlled by how well one rejects heat to the cold sink. Combining Equations (10), (12), (14) and (16), we obtain

(17) $$\frac{T_{1}-E_{L}T_{L}}{1-E_{L}}=\left(1-\eta_{E}\right)\left[T_{1}+E_{H}\left(T_{H}-T_{1}\right)\right]+\eta_{E}{T_{1}\left(\frac{T_{1}+E_{H}\left(T_{H}-T_{1}\right)}{T_{1}}\right)}^{\frac{1}{k}}.$$

Now, we can calculate the heat flows; for process 1→2, the fluid absorbs heat from the hot source, and then we obtain

(18) $${\dot{Q}}_{12}=\dot{m}C_{v}E_{H}\left(T_{H}-T_{1}\right)={\dot{m}C}_{v}\left(T_{2}-T_{1}\right).$$

Whereas for process 3→1, the fluid gives up heat to the cold source, so we can write

(19) $${\dot{Q}}_{31}=\dot{m}C_{p}E_{L}\left(T_{3}-T_{L}\right)=\dot{m}C_{p}\left(T_{3}-T_{1}\right).$$

By combining the previous equation with Equation (16), we obtain

(20) $${\dot{Q}}_{31}=\dot{m}C_{p}\frac{E_{L}}{1-E_{L}}\left(T_{1}-T_{L}\right)=\dot{m}kC_{v}\frac{E_{L}}{1-E_{L}}\left(T_{1}-T_{L}\right).$$

The net power output of the steady-state cycle can be obtained from the first law of thermodynamics; that is

(21) $$P=\dot{W}={\dot{Q}}_{12}-{\dot{Q}}_{31}.$$

Substituting the heat flow expressions, which are Equations (18)–(20), we have

(22) $$P=\dot{m}C_{v}\left[E_{H}\left(T_{H}-T_{1}\right)-k\frac{E_{L}}{1-E_{L}}\left(T_{1}-T_{L}\right)\right].$$

We can now write the efficiency in terms of temperatures and effectiveness, which is given by

(23) $$\eta =\frac{P}{{\dot{Q}}_{12}}=1-\frac{kE_{L}\left(T_{1}-T_{L}\right)}{E_{H}\left(1-E_{L}\right)\left(T_{H}-T_{1}\right)}.$$

### 2.4. Entropy Production and the Origin of Thermal Irreversibility

In addition to energy, we need to characterize irreversibilities. In this cycle, we assume that all relevant dissipative behavior comes from two sources: (i) the finite temperature difference in the heat exchangers and (ii) the internal irreversibility of the expansion, already captured by $\eta_{E}.$

If we consider only the heat exchangers, we can estimate the total entropy production ${\dot{S}}_{gen}$ associated with heat transfer between the fluid and the thermal reservoirs. To perform this, we model that, in the hot heat exchanger, the hot reservoir loses heat ${\dot{Q}}_{12}$ at a nearly constant temperature $T_{H}$, while the fluid gains heat, but at an effective mean temperature ${\bar{T}}_{12}$, which we approximate as

(24) $${\bar{T}}_{12}=\frac{T_{1}+T_{L}}{2}.$$

Similarly, in the cold exchanger, the fluid discharges heat ${\dot{Q}}_{31}$ at an average temperature.

(25) $${\bar{T}}_{31}=\frac{T_{1}+T_{3}}{2}.$$

The cold reservoir receives it at $T_{L}$, which we assume to be constant

(26) $${\dot{S}}_{gen}={\dot{Q}}_{12}\left(\frac{1}{{\bar{T}}_{12}}-\frac{1}{T_{L}}\right)+{\dot{Q}}_{31}\left(\frac{1}{T_{L}}-\frac{1}{{\bar{T}}_{31}}\right)\geq 0.$$

This equation shows the actual heat transfer of a reversible transfer and gives us a quantity that we can later combine with power to build “eco-efficiency” metrics that have physical meaning (e.g., how much power we obtain per unit of irreversibility).

## 3. Objective Functions

So far, we have only discussed power and thermal efficiency, which are classical metrics. But now, we want to incorporate the entropic cost, that is, the irreversibilities generated in the cycle, especially in the heat exchangers. Recall that the total rate of entropy generation that we attribute to the exchangers is given by Equations (24)–(26), which give us information about how far we are from the reversible ideal. If the exchangers were infinitely large and there was no temperature difference between fluids and reservoirs, ${\dot{S}}_{gen}$ would tend to be zero. On this basis, we define three useful operating regimes.

### 3.1. Bounded Ecological Function Regime

Some of the literature on irreversible cycles uses the so-called “ecological function” ($E_{F}=P-T_{L}{\dot{S}}_{gen}$), but this function can become negative if losses due to irreversibility are large. To avoid this sign ambiguity, we propose using the positive dimensionless version.

(27) $$\phi =\frac{P}{P+T_{L}{\dot{S}}_{gen}}.$$

This quantity is between 0 and 1; ϕ is always positive, always bounded, and always easy to compare. The closer it is to 1, the more of the “total available resource” (which, in this case, we interpret as useful power plus equivalent entropic destruction power measured at $T_{L}$) ends up as useful power and not as irreversibility.

### 3.2. Second Law Efficiency

Another common approach in exergy analysis is to compare the power generated with the exergy available in the absorbed heat, $B_{in}$. If the hot source is modeled as a reservoir at a fixed temperature $T_{H}$, the thermal exergy supplied by ${\dot{Q}}_{12}$ can be approximated as

(28) $$B_{in}\approx \left(1-\frac{T_{L}}{T_{H}}\right){\dot{Q}}_{12},$$

where $T_{L}$ is the reference cold temperature. We define the second law efficiency by

(29) $$\eta_{II}=\frac{P}{B_{in}}=\frac{\dot{m}C_{v}\left[E_{H}\left(T_{H}-T_{1}\right)-k\frac{E_{L}}{1-E_{L}}\left(T_{1}-T_{L}\right)\right]}{\left(1-\frac{T_{L}}{T_{H}}\right){\dot{Q}}_{12}}.$$

This quantity compares the actual power generated with the available thermal exergy from the heat absorbed from the hot source, and this quantity ranges between 0 and 1.

In the $\phi \left(u_{L}\right)$ and $\eta_{II}\left(u_{L}\right)$ graphs, we again observe well-defined maxima. Most interestingly, the $u_{L}$ values that maximize ϕ and $\eta_{II}$ tend to be close to the value that maximizes P. This is extremely convenient from a design perspective; it means we are not forced to sacrifice almost all our power just to improve our “ecological quality” or our exergy efficiency. Generally, there is a reasonable compromise zone around a given $u_{L}$ distribution.

### 3.3. Efficient Power Regime

In addition to P and η, in irreversible cycle thermodynamics, efficient power is frequently used, which is defined as follows:

(30) $$E_{P}=P\eta .$$

This objective function optimizes power to achieve maximum performance with minimum energy consumption, thus minimizing losses and waste. Substituting Equations (22) and (23) into the previous equation and simplifying, we obtain the following:

(31) $$E_{P}=\left(\dot{m}C_{v}\left[E_{H}\left(T_{H}-T_{1}\right)-k\frac{E_{L}}{1-E_{L}}\left(T_{1}-T_{L}\right)\right]\right)\left(1-\frac{kE_{L}\left(T_{1}-T_{L}\right)}{E_{H}\left(1-E_{L}\right)\left(T_{H}-T_{1}\right)}\right).$$

When $P_{E}\left(u_{L}\right)$ is plotted, a maximum also appears. And again, this maximum usually falls in a region very close to the one that maximizes P and maximizes ϕ. This consistency between different criteria (gross power P, efficient power $E_{P}$, bounded ecological efficiency ϕ, second law efficiency $\eta_{II}$) is exactly what one wants to see from a design perspective. It indicates that there is a physically relevant range of $u_{L}$, where we not only obtain a lot of power but we obtain it in a reasonably efficient way and with bounded energy quality destruction.

## 4. Numerical Results

This section presents and analyzes the numerical results for the irreversible steady flow Lenoir cycle. External heat transfer irreversibilities in the hot and cold exchangers are modeled through the effectivenesses ($E_{H},E_{L}$) derived from an ε−NTU framework, while the internal irreversibility of the expansion is represented by a finite isentropic efficiency $\eta_{E}$. To allow a direct comparison with previous studies, we adopt parameter values similar to those used by Rubio and Wang [38] and in related works [26,34,35,36,37]. The working fluid is assumed to be air with constant properties, namely, a specific heat at a constant volume $C_{v}=0.7165\;\mathrm{kJ}/(\mathrm{kg}\cdot K)$ and a specific heat ratio k=1.4. The isentropic efficiency of the gas turbine is taken as $\eta_{E}=0.92$. Unless otherwise stated, the mass flow rate and the cold reservoir temperature are fixed at $\dot{m}=1.1165\;\mathrm{kg}/s$ and $T_{L}=320\;K$, respectively.

**Figure 2:**
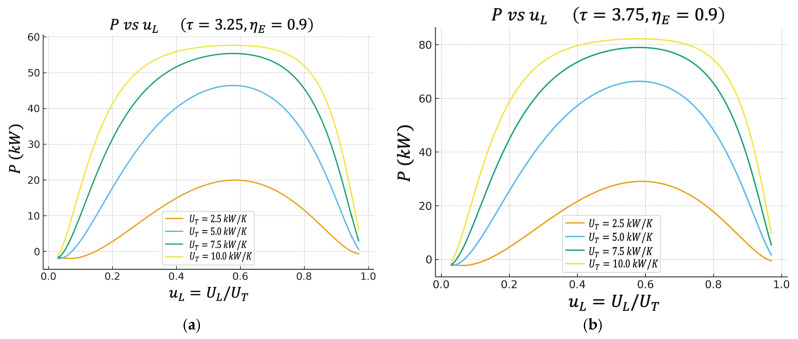
(**a**) Effect of $U_{T}$ on $P\;\mathrm{vs}.\;u_{L}$ characteristics when τ=3.25. (**b**) Effect of $U_{T}$ on $P\;\mathrm{vs}.\;u_{L}$ characteristics when τ=3.75.

**Figure 3:**
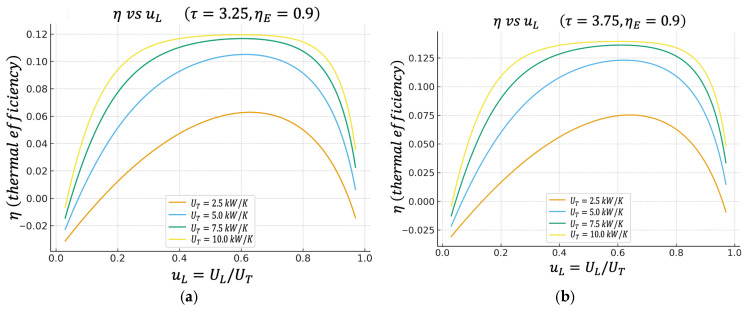
(**a**) Effect of $U_{T}$ on $\eta \;\mathrm{vs}.\;u_{L}$ characteristics when τ=3.25. (**b**) Effect of $U_{T}$ on $\eta \;\mathrm{vs}.\;u_{L}$ characteristics when τ=3.75.

**Figure 4:**
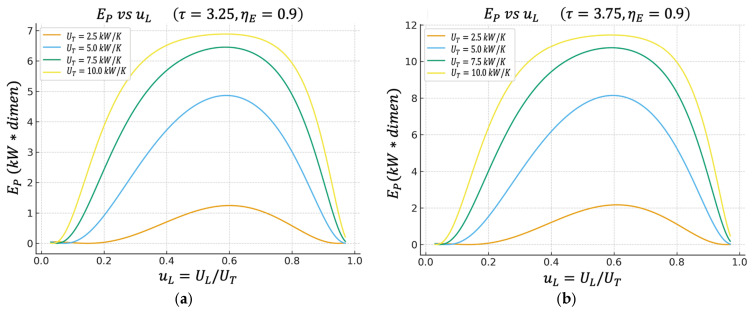
(**a**) Effect of $U_{T}$ on $\;E_{P}\;\mathrm{vs}.\;u_{L}$ characteristics when τ=3.25. (**b**) Effect of $U_{T}$ on $E_{P}\;\mathrm{vs}.\;u_{L}$ characteristics when τ=3.75.

**Figure 5:**
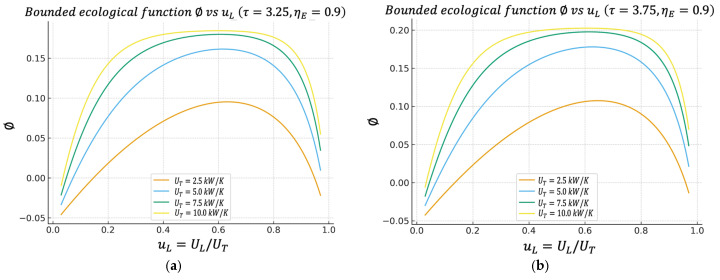
(**a**) Effect of $U_{T}$ on $\phi \;\mathrm{vs}.\;u_{L}$ characteristics when τ=3.25. (**b**) Effect of $U_{T}$ on $\phi \;\mathrm{vs}.\;u_{L}$ characteristics when τ=3.75.

**Figure 6:**
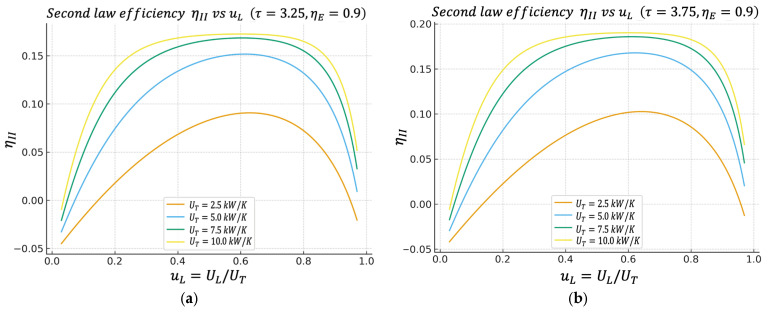
(**a**) Effect of $U_{T}$ on $\eta_{II}\;\mathrm{vs}.\;u_{L}$ characteristics when τ=3.25. (**b**) Effect of $U_{T}$ on $\eta_{II}\;\mathrm{vs}.\;u_{L}$ characteristics when τ=3.75.

**Figure 7:**
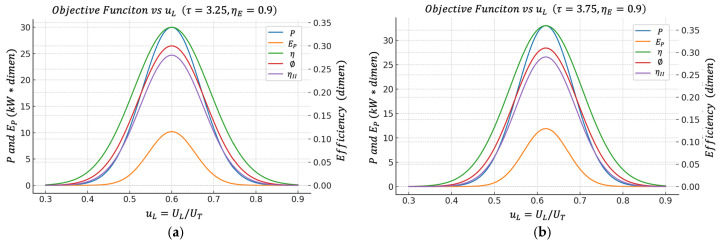
(**a**) Effect of $U_{T}$ on the objective $\mathrm{vs}.\;u_{L}$ functions when τ=3.25. (**b**) Effect of $U_{T}$ on the objective functions $\mathrm{vs}.\;u_{L}$ when τ=3.75.

Unless otherwise stated, all performance maps shown in Figure 2, Figure 3, Figure 4, Figure 5, Figure 6 and Figure 7 are computed using this same baseline data. The overall thermal conductance $U_{T}$ varies within the range indicated in the captions, and the conductance–allocation parameter φ distributes $U_{T}$ between the hot and cold exchangers. Figure 2 and Figure 3 display the behaviour of the net power and the pure thermal efficiency under these parametric sweeps, while Figure 4, Figure 5 and Figure 6 show the corresponding responses of the efficient power, the bounded ecological function, and the second law efficiency, respectively. Figure 7 summarizes and compares the optimal values of φ obtained from these different objective functions.

### 4.1. Net Power (P) vs. Fraction of Conductance on the Cold Side

The figure shows the net power P as a function of $u_{L}=U_{L}/U_{T}$ for different values of $U_{T}$ in two cases of the temperature ratio τ: (a) τ=3.25 and (b) τ=3.75. From Figure 2a,b, we can observe that $P\left(u_{L}\right)$ has a bell shape; for very low values of $u_{L}$, the power is reduced, increases to a clear maximum, and then falls again when $u_{L}$ gets too close to 1.

This maximum means that it is not advisable to allocate all the thermal conductance to the heater or all the thermal conductance to the cooler; there is an optimal distribution.

When $U_{T}$ increases (for example, from 2.5 kW/K to 10 kW/K), the height of the curve increases; the cycle can deliver more kW of total net power. Furthermore, the region near the maximum widens, implying that the cycle becomes more tolerant of deviations from $u_{L}$.

This reproduces the type of behavior reported in classical analyses of the irreversible Lenoir cycle. There is an optimal allocation of area/conductance between the two heat exchange stages that maximizes the useful power delivered by the engine.

### 4.2. Thermal Efficiency η Versus $u_{L}$

Figure 3a,b show the thermal efficiency $\eta =P/{\dot{Q}}_{12}$ of the cycle as a function of $u_{L}$, again for two cases of the temperature ratio τ, (a) τ=3.25 and (b) τ=3.75, and for the same values of $U_{T}$.

The results show that $\eta \left(u_{L}\right)$ also has a well-defined maximum. The value of $u_{L}$ that maximizes η is very close, although not exactly at the same point, to the value of $u_{L}$ that maximizes the power P. This observation provides a direct and quantitative characterization of the inherent trade-off between power output and thermal efficiency in real thermal engines. Specifically, operating the cycle precisely at the point of maximum power yields a thermal efficiency that, although not maximal, remains relatively high—often within a few percentage points of the maximum attainable value. Conversely, shifting the operation toward the point of maximum thermal efficiency results in only a moderate reduction in power output, not a dramatic loss as might be expected in more sharply peaked systems.

### 4.3. Efficient Power Regime Versus $u_{L}$

Figure 4a,b introduce the efficient power objective function $E_{P}=P\eta$, which is designed for practical engineering and thermo-economic evaluation, rewarding states where the power is large and, at the same time, the thermal efficiency is not ridiculously low.

In the following figure, it can be observed that the maximum of $E_{P}$ appears practically in the same region of $u_{L}$ that maximizes the net power P.

This indicates that the optimal power operating point also has attractive performance when simultaneously requiring “how much power I get out” and “how well I convert the heat from the hot source into useful work.”

### 4.4. Bounded Ecological Function Regime Versus $u_{L}$

Figure 5a,b show the so-called bounded ecological efficiency ϕ, defined by Equation (27). This definition is positive compared to other formulations, since it is the definition of the classical ecological function. If one defines the function as $E_{F}=P-T_{L}{\dot{S}}_{gen}$, then this quantity can become negative in very dissipative regimes (because the entropic penalty dominates), which sometimes makes direct physical interpretation difficult.

The figure shows that $\phi \left(u_{L}\right)$ also exhibits a maximum value, which again falls within the same band as $P\left(u_{L}\right)$ and $P_{E}\left(u_{L}\right)$, where they have maxima. This means that the conductance assignment that maximizes the cycle’s power does not generate disproportionate exergy destruction. From an environmental/second law perspective, the power optimum is acceptable. This is particularly significant, as it offers both a physical and environmental interpretation of the optimal operating point. The system not only achieves high power but also maintains thermodynamically efficient behavior, with moderate levels of internal irreversibility.

### 4.5. Second Law Efficiency Regime Versus $u_{L}$

Figure 6a,b show the behavior of the second law efficiency regime $\eta_{II}$ given by Equation (29). Like the previous behaviors, $\eta_{II}\left(u_{L}\right)$ has a maximum located in the $u_{L}$ region, which is similar to the other previous regimes.

This result confirms the argument: the same conductance distribution range between the hot and cold heat exchangers that is optimal for power output is also very good from the perspective of overall exergy utilization. This is important when justifying design under second law criteria or energy/industrial audits, because high power output alone is not enough; it must be demonstrated that the exergy from the hot reservoir is not wasted.

## 5. Conclusions

Finally, we constructed Table 1 and Table 2, which show a summary listing; for each combination ($\tau ,U_{T}$), the value of $u_{L}$ that maximizes each objective function includes net power P, thermal efficiency η, efficient power $E_{P}$, bounded ecological efficiency ∅, and second law efficiency $\eta_{II}$. The corresponding maximum values are also reported (e.g., $P^{*}$ in kW, $\eta^{*}$, and similarly for the other regimes). From the results shown in the table above, we can draw the following conclusions.

1. For each τ and each $U_{T}$, the optimal value of $u_{L}$ that maximizes P is typically around $u_{L}\approx 0.6$ (for example, in the case of τ=3.25 and $U_{T}=2.5\;kW/K$, an optimum was obtained around $u_{L}≅0.59$, with maximum power on the order of tens of kW, efficiency η~0.2, and so on). These results can be clearly seen in Figure 7.

2. The $u_{L}$ that maximizes η (pure thermal efficiency), the one that maximizes $E_{P}$, the one that maximizes ∅, and the one that maximizes $\eta_{II}$ are all extremely close to this same optimal power value.

3. When $U_{T}$ increases (for example, from 2.5 kW/K to 10.0 kW/K), the maximum power $P^{*}$ increases appreciably, but the optimum zone of $u_{L}$ remains approximately in the same band. That is, providing the system with more heat exchange capacity improves the quantitative efficiency (more kW) but does not drastically change the conductance distribution strategy between the hot (1→2) and cold (3→1) heat exchangers.

From an engineering point of view, this has a very useful interpretation since there is a robust operating band around $u_{L}\sim 0.6$ that simultaneously maximizes net power, maintains high thermal efficiency, maximizes efficient power, minimizes relative entropic impact (high ∅), and maximizes exergy utilization (high $\eta_{II}$).

This convergence between classical regimes (power, efficiency) and more ambient or second law regimes (∅,$\;\eta_{II}$) is one of the strongest results of this study because it suggests that the design optimum does not critically depend on choosing a single arbitrary figure of merit. Instead, several reasonable figures of merit point to the same design range, making this recommendation more defensible in real-world applications (for example, in waste heat recovery in industrial plants, where useful power is of interest but overall exergy performance is also monitored). Instead, several reasonable figures of merit point to the same design range, making this recommendation more defensible in real-world applications, such as waste heat recovery units in industrial plants, pulse combustion or pulsed-detonation engines, and small gas turbine or micro-CHP systems, where not only useful power but also exergy performance and environmental impact are monitored.

## Figures and Tables

**Table 1:** Numerical results for the different operating regimes ($P^{*},\eta^{*}$) and effectiveness.

τ	$U_{T}$	$\eta_{E}$	$u_{L}^{*}$	$P^{*}$	$u_{L}^{*}$	$\eta^{*}$	$u_{L}^{*}$
	(kW/K)		(P)	(kW)	$\left(\eta \right)$		$\left(E_{P}\right)$
3.25	2.5	0.9	0.584	19.928	0.627	0.062	0.601
3.25	5	0.9	0.575	46.394	0.614	0.105	0.592
3.25	7.5	0.9	0.579	55.342	0.605	0.117	0.588
3.25	10	0.9	0.579	57.625	0.601	0.120	0.588
3.75	2.5	0.9	0.588	29.057	0.640	0.075	0.610
3.75	5	0.9	0.578	66.340	0.622	0.123	0.597
3.75	7.5	0.9	0.579	78.950	0.610	0.136	0.592
3.75	10	0.9	0.579	82.167	0.610	0.139	0.588

**Table 2:** Numerical results for the different operating regimes ($E_{P}^{*},\emptyset^{*},\eta_{II}^{*}$) and effectiveness.

τ	$U_{T}$	$\eta_{E}$	$E_{P}^{*}$	$u_{L}^{*}$	$\emptyset^{*}$	$u_{L}^{*}$	$\eta_{II}^{*}$
	(kW/K)			$\left(\emptyset \right)$		$\left(\eta_{II}\right)$	
3.25	2.5	0.9	1.244	0.631	0.095	0.627	0.091
3.25	5	0.9	4.864	0.618	0.161	0.614	0.152
3.25	7.5	0.9	6.453	0.605	0.180	0.605	0.169
3.25	10	0.9	6.885	0.601	0.184	0.601	0.173
3.75	2.5	0.9	2.168	0.644	0.107	0.640	0.103
3.75	5	0.9	8.141	0.627	0.178	0.622	0.168
3.75	7.5	0.9	10.747	0.614	0.198	0.609	0.186
3.75	10	0.9	11.454	0.605	0.203	0.605	0.190

## Data Availability

The data that support the findings of this study are available from the corresponding author upon request.
